# The Global Burden of Disease Study 2010: Interpretation and Implications for the Neglected Tropical Diseases

**DOI:** 10.1371/journal.pntd.0002865

**Published:** 2014-07-24

**Authors:** Peter J. Hotez, Miriam Alvarado, María-Gloria Basáñez, Ian Bolliger, Rupert Bourne, Michel Boussinesq, Simon J. Brooker, Ami Shah Brown, Geoffrey Buckle, Christine M. Budke, Hélène Carabin, Luc E. Coffeng, Eric M. Fèvre, Thomas Fürst, Yara A. Halasa, Rashmi Jasrasaria, Nicole E. Johns, Jennifer Keiser, Charles H. King, Rafael Lozano, Michele E. Murdoch, Simon O'Hanlon, Sébastien D. S. Pion, Rachel L. Pullan, Kapa D. Ramaiah, Thomas Roberts, Donald S. Shepard, Jennifer L. Smith, Wilma A. Stolk, Eduardo A. Undurraga, Jürg Utzinger, Mengru Wang, Christopher J. L. Murray, Mohsen Naghavi

**Affiliations:** 1 National School of Tropical Medicine at Baylor College of Medicine, Houston, Texas, United States of America; 2 Sabin Vaccine Institute and Texas Children's Hospital Center for Vaccine Development, Houston, Texas, United States of America; 3 James A. Baker III Institute at Rice University, Houston, Texas, United States of America; 4 Institute for Health Metrics and Evaluation, University of Washington, Seattle, Washington, United States of America; 5 Imperial College London, London, United Kingdom; 6 Vision and Eye Research Unit, Anglia Ruskin University, Cambridge, United Kingdom; 7 Institut de Recherche pour le Développement, Montpellier, France; 8 London School of Hygiene and Tropical Medicine, London, United Kingdom; 9 Inovio Pharmaceuticals, Inc., Blue Bell, Pennsylvania, United States of America; 10 Johns Hopkins University Bloomberg School of Public Health, Baltimore, Maryland, United States of America; 11 Texas A&M University, College Station, Texas, United States of America; 12 University of Oklahoma Health Sciences Center, Oklahoma City, Oklahoma, United States of America; 13 Erasmus MC, University Medical Center Rotterdam, Rotterdam, Netherlands; 14 Institute of Infection and Global Health, University of Liverpool, Liverpool, United Kingdom; 15 International Livestock Research Institute, Nairobi, Kenya; 16 Swiss Tropical and Public Health Institute, Basel, Switzerland; 17 University of Basel, Basel, Switzerland; 18 Brandeis University, Waltham, Massachusetts, United States of America; 19 Case Western Reserve University, Cleveland, Ohio, United States of America; 20 Watford General Hospital, Watford, United Kingdom; 21 Vector Control Research Centre, Pondicherry, India; 22 Stanford University School of Medicine, Stanford, California, United States of America; University of Kelaniya, Sri Lanka

## Introduction

The publication of the Global Burden of Disease Study 2010 (GBD 2010) and the accompanying collection of *Lancet* articles in December 2012 provided the most comprehensive attempt to quantify the burden of almost 300 diseases, injuries, and risk factors, including neglected tropical diseases (NTDs) [1]–[3]. The disability-adjusted life year (DALY), the metric used in the GBD 2010, is a tool which may be used to assess and compare the relative impact of a number of diseases locally and globally [4]–[6]. Table 1 lists the major NTDs as defined by the World Health Organization (WHO) [7] and their estimated DALYs [1]. With a few exceptions, most of the NTDs currently listed by the WHO [7] or those on the expanded list from *PLOS Neglected Tropical Diseases* [8] are disablers rather than killers, so the DALY estimates represent one of the few metrics available that could fully embrace the chronic effects of these infections.

**Table 1:** Estimated DALYs (in millions) of the NTDs from the Global Burden of Disease Study 2010.

Disease	DALYs from GBD 2010 (numbers in parentheses indicate 95% confidence intervals) [1]
**NTDs**	26.06 (20.30–35.12)
**Intestinal nematode infections**	5.19 (2.98–8.81)
Hookworm disease	3.23 (1.70–5.73)
Ascariasis	1.32 (0.71–2.35)
Trichuriasis	0.64 (0.35–1.06)
**Leishmaniasis**	3.32 (2.18–4.90)
**Schistosomiasis**	3.31 (1.70–6.26)
**Lymphatic filariasis**	2.78 (1.8–4.00)
**Food-borne trematodiases**	1.88 (0.70–4.84)
**Rabies**	1.46 ((0.85–2.66)
**Dengue**	0.83 (0.34–1.41)
**African trypanosomiasis**	0.56 (0.08–1.77)
**Chagas disease**	0.55 (0.27–1.05)
**Cysticercosis**	0.50 (0.38–0.66)
**Onchocerciasis**	0.49 (0.36–0.66)
**Trachoma**	0.33 (0.24–0.44)
**Echinococcosis**	0.14 (0.07–0.29)
**Yellow fever**	<0.001
**Other NTDs** *	4.72 (3.53–6.35)

* Relapsing fevers, typhus fever, spotted fever, Q fever, other rickettsioses, other mosquito-borne viral fevers, unspecified arthropod-borne viral fever, arenaviral haemorrhagic fever, toxoplasmosis, unspecified protozoal disease, taeniasis, diphyllobothriasis and sparganosis, other cestode infections, dracunculiasis, trichinellosis, strongyloidiasis, enterobiasis, and other helminthiases.

Even DALYs, however, do not tell the complete story of the harmful effects from NTDs. Some of the specific and potential shortcomings of GBD 2010 have been highlighted elsewhere [9]. Furthermore, DALYs measure only direct health loss and, for example, do not consider the economic impact of the NTDs that results from detrimental effects on school attendance and child development, agriculture (especially from zoonotic NTDs), and overall economic productivity [10], [11]. Nor do DALYs account for direct costs of treatment, surveillance, and prevention measures. Yet, economic impact has emerged as an essential feature of the NTDs, which may trap people in a cycle of poverty and disease [10]–[12]. Additional aspects not considered by the DALY metrics are the important elements of social stigma for many of the NTDs and the spillover effects to family and community members [13], [14], loss of tourism [15], and health system overload (e.g., during dengue outbreaks). Ultimately NTD control and elimination efforts could produce social and economic benefits not necessarily reflected in the DALY metrics, especially among the most affected poor communities [11].

## Variations in DALYs

Despite the importance of the concept of disease burden and disability to the NTD community, assigning DALYs or related metrics to each NTD has been a bit of a roller-coaster ride over the past decade and may continue to be for many years to come. Significant variations in ascribing DALYs to the NTDs are due to many factors, including data scarcity and inherent difficulties in accurately estimating the number of individuals at risk, the number of incident cases, the number of prevalent cases, and, among these, the duration of the infection. Challenges also include uncertainty about the relationship between acute and chronic infections and their link to specific morbidities, duration of morbidity, and the proportion of the population infected or with morbidities that are treated versus untreated. An additional challenge is to obtain all of the aforementioned values stratified by age and gender, data which are seldom available for NTDs. Moreover, the affordable diagnostic tools typically used to measure NTDs in resource-constrained settings are inaccurate and many sequelae (i.e., morbidities) of NTDs are nonspecific, making it difficult to attribute them to a particular infection or risk factor. For several NTDs, controversies remain regarding what proportion of a sequelae should be ascribed to different infections or diseases. An extreme example is the case of schistosomiasis, for which disease burden estimates over the past decade have ranged from 1.7 million DALYs to as many as 56 million DALYs, depending on whether higher disease prevalence estimates are considered and if specific chronic morbidities are attributed to this NTD [12]. The variation is also due to continuous refinement of definitions and methodologies for burden estimation, which affects the estimates for all diseases, injuries, and risk factors and further complicates the comparison of different GBD versions. Among the furthest-reaching methodological alterations of GBD 2010 are the shift from incidence- to prevalence-based DALYs, the abandonment of age weighting and discounting, the application of refined reference life tables and disability weights, and the introduction of comorbidity adjustments [16].

Some of the greatest variation in the disease burden estimates over the past decade has been observed among the three major intestinal nematode infections (also known as soil-transmitted helminthiases, i.e., ascariasis, hookworm disease, and trichuriasis) as well as in schistosomiasis. A key reason for this wide variation is the fact that these helminth infections are among the most common infections of humankind [17]–[19], so small variations in an assigned disability weight become amplified by the hundreds of millions of people estimated to harbor these parasites. Another reason for variations in some burden estimates is due to how GBD 2010 uniquely classified certain diseases or groups of diseases. A prominent example was the decision to combine the burdens of cystic echinococcosis and alveolar echinococcosis into a single estimate (i.e., echinococcosis). This was a questionable decision seeing that the two parasites have different life cycles, geographic distributions, and clinical outcomes. Future iterations of the GBD will therefore need to consider reporting these estimates as separate conditions, paying greater attention to the unique attributes of the individual parasites.

Overall, the NTD community was dismayed by the previous WHO estimates between 1999 and 2004 [20], which assigned DALYs that were equivalent to conditions of comparatively minor global health importance for major diseases such as schistosomiasis [21]. At the other extreme, the higher DALY estimates for NTDs elevate the status of these diseases to a level at which they could be thought of as the fourth leg to a table built on HIV/AIDS, tuberculosis, and malaria [22]. The GBD 2010 is an ambitious attempt to resolve some of the differences between earlier estimates (including use of strictly comparable data and methods for 1990, 2005, and 2010) and to provide a first attempt at estimating the disease burden of cysticercosis, echinococcosis, and rabies as part of the largest ever burden of disease study [1]–[3]. The GBD 2010 also provides first-time disease burden estimates for amebiasis, cryptosporidiosis, trichomoniasis, scabies, fungal skin infections, and venomous animal contact (including snake bite), although they are not listed under the NTD category (Table 2) [1]–[3]. One surprising finding from these estimates was the huge disease burden that results from cryptosporidiosis among young children. Together, the NTDs listed in Table 1 and those in Table 2 add up to almost 48 million DALYs. This number is comparable to tuberculosis (49 million) and is more than half of the global burden of two of the world's major diseases, malaria (83 million) and HIV/AIDS (82 million). However, these comparisons must be conducted with great care given the large variation in the quantity and quality of epidemiological data currently available across the world.

**Table 2:** Other NTDs in the Global Burden of Disease Study 2010 not listed in the “NTD and malaria” category.^1^

Disease	DALYs from GBD 2010 in millions (numbers in parentheses indicate 95% confidence intervals) [1]
**Cryptosporidiosis**	8.37 (6.52–10.35)
**Cholera**	4.46 (3.34–5.80)
**Animal contact (venomous)**	2.72 (1.54–4.80)
**Amebiasis**	2.24 (1.73–2.84)
**Fungal skin diseases**	2.30 (0.72–5.27)
**Scabies**	1.58 (0.80–2.79)
**Trichomoniasis**	0.17 (0.01–0.53)
**Leprosy**	0.006 (0.002–0.11)
**Total**	21.84
**Total of NTDs in ** **Table 1** ** (from GBD 2010) and NTDs in ** **Table 2**	47.90

1 The table provides numbers of DALYs in millions as calculated in GBD 2010 [1]. The diseases are not listed as NTDs in GBD 2010 and, with the exception of leprosy, these diseases are also not on the WHO list of 17 NTDs [5]. However, these conditions (as well as some other diarrheal diseases) are considered by *PLOS Neglected Tropical Diseases* [6].

## Killers and Disablers

**Figure 1:**
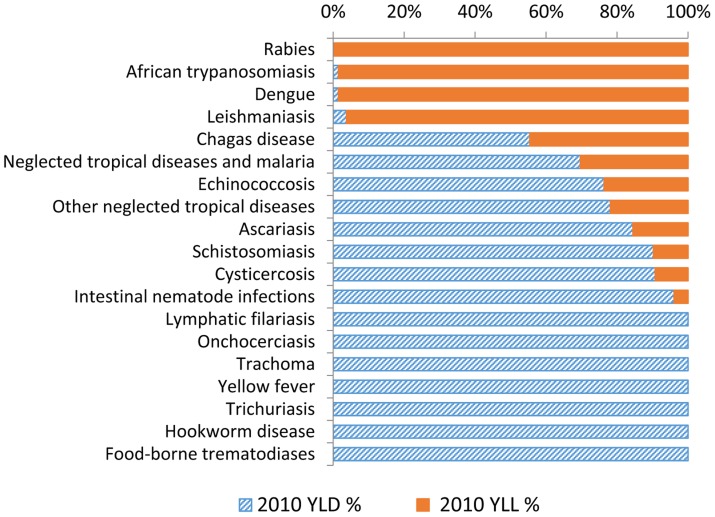
Fractions of YLD and YLL (as components of DALYs) for each of the NTDs. Also included in this graph are “other NTDs.”

Some of the details of the new disease burden estimates for NTDs are summarized in Table 3, while the total number of estimated cases is summarized in Table 4. Briefly, as stated by Murray et al. (2012), “DALYs are the sum of two components: years of life lost due to premature mortality (YLLs) and years lived with disability (YLDs)” [1]. For many of the major NTDs, including hookworm disease and the other intestinal nematode infections, schistosomiasis, food-borne trematodiases, onchocerciasis, cysticercosis, and trachoma, most (and in some cases all) of the reported DALYs result from YLDs (i.e., disability, not deaths) (Figure 1). These NTDs are genuinely not thought of as killer diseases, although it has been noted that some disabling NTDs such as onchocerciasis, cysticercosis, and food-borne trematodiases cause excess mortality associated with blindness, heavy infection in sighted individuals, hydrocephalus, stroke, gliomas, ectopic infections, cholangiocarcinoma, and other (yet unmeasured) factors [23]–[26]. An added feature about the publication of the YLDs from the NTDs was the listing of the specific sequelae that were considered in deriving these estimates [3], which allows comparability across studies.

**Table 3:** DALYs, YLDs, YLLs, and deaths from NTDs from the Global Burden of Disease Study.

Disease	DALYs in millions [1]	DALY rank	YLDs in millions [3]	YLD rank	YLLs in millions [1]–[3]	YLL rank	Deaths [2]	Death rank
**All NTDs**	26.06		18.22		7.90		152,000	
**Intestinal nematode infections**	5.19	1	4.98	1	0.20	7	2,700	7
Hookworm disease	3.23	4	3.23	2	0	-	-	
Ascariasis	1.32	8	1.11	6	0.20	7	2,700	7
Trichuriasis	0.64	10	0.64	7	0	-	-	
**Leishmaniasis**	3.32	2	0.12	12	3.19	1	51,600	1
**Schistosomiasis**	3.31	3	2.99	3	0.32	5	11,700	4
**Lymphatic filariasis**	2.78	5	2.77	4	0	-	-	
**Food-borne trematodiases**	1.88	6	1.87	5	0	-	-	
**Rabies**	1.46	7	<0.01	16	1.46	2	26,400	2
**Dengue**	0.83	9	0.01	15	0.81	3	14,700	3
**African trypanosomiasis**	0.56	11	0.08	14	0.55	4	9,100	6
**Chagas disease**	0.55	12	0.30	11	0.24	6	10,300	5
**Cysticercosis**	0.50	13	0.46	9	0.05	8	1,200	8
**Onchocerciasis**	0.49	14	0.49	8	0	-	-	
**Trachoma**	0.33	15	0.33	10	0	-	-	
**Echinococcosis**	0.14	16	0.11	13	0.03	9	1,200	8
**Yellow fever**	<0.001	17	<0.01	16	<0.01	10	-	
**Other NTDs**	4.72	-	3.69	-	1.03	-	23,700	

**Table 4:** Expected number of cases in 2010 and 95% confidence intervals of the neglected tropical diseases (mean and uncertainty) as extrapolated from the Global Burden of Disease Study 2010.

Disease	Number of cases	95% confidence intervals	Selected comments
Ascariasis^1^	819 million	772–892 million	Total number of cases
Trichuriasis^1^	465 million	430–508 million	Total number of cases
Hookworm disease^1^	439 million	406–480 million	Total number of cases
Schistosomiasis	252 million	252–252 million	Total number of cases
Onchocerciasis	30.4 million	27.3–33.6 million	Total number of cases with adult worms*
Lymphatic filariasis	36 million	34–39 million	Lymphedema and/or hydrocele only
Food-borne trematodiases	16 million	7–41 million	Heavy and cerebral infections only
Cutaneous leishmaniasis	10 million	8–13 million	Total number of cases
Chagas disease	7.5 million	2.5–12.4 million	Symptomatic cases only
Trachoma	4.4 million	3.5–5.5 million	Low vision and blindness cases only
Cysticercosis	1.4 million	1.3–1.6 million	Epilepsy cases only
Echinococcosis	1.1 million	0.6–2.1 million	Symptomatic liver, lung, and central nervous system cases only
Dengue	179,000 cases	109,000–299,000	Incident (acute) symptomatic cases only
Visceral leishmaniasis	76,000 cases	61,000–93,500	Total number of cases
African trypanosomiasis	37,000 cases	9,000–106,000	Symptomatic cases only
Rabies	1,100 cases	600–2,000	Incident cases
Yellow fever	100 cases	0–100 cases	Incident cases

* This number includes 14.6 million people (13.2–16.1 million) with detectable skin microfilariae.

1 These are updated estimates recently published in Pullan et al. [27].

According to the GBD 2010 estimations, intestinal nematode infections rank first in the list of the NTDs for which a DALY was estimated [27]. Among intestinal nematodes, hookworm disease was estimated as having the largest YLDs (and 62% of the DALYs). This large contribution of hookworm disease to the YLDs of nematodes comes from the inclusion of recent information linking hookworm disease to moderate and severe anemia across several different populations, including children and pregnant women [28], [29]. On the other hand, important comorbidity effects resulting from hookworm disease and malaria coinfections [30]–[32] and the deaths from these conditions were attributed to malaria in the GBD 2010, reducing the apparent YLLs of hookworm infections.

Schistosomiasis was estimated to rank second in terms of YLDs (and right behind the intestinal nematode infections in terms of prevalence). Schistosomiasis was one of the NTDs that generated the most controversy and debate in the GBD 2010. Since 2005, important information has been generated about the effects of schistosomiasis that result in chronic pain, inflammation, malnutrition, and exercise intolerance, among other morbid sequelae [12], [21], [33], which under some scenarios generated DALY estimates that exceeded those of malaria or other better-known conditions [12]. However, many of these aspects were not accepted into the GBD 2010, in part because of disagreements about the long-term health importance and actual YLLs caused by these elements. Fueling the schistosomiasis controversy even further were previously published annual mortality estimates for schistosomiasis (i.e., 280,000 in Africa alone) [33] suggesting that the number of people killed from this disease was at least 20 times higher than indicated in GBD 2010 [34]. In addition, there is new information on the links between female urogenital schistosomiasis and the risk of acquiring HIV/AIDS [35]. The discussions surrounding the burden of schistosomiasis may just be the start of future investigations on how to best attribute parts of the burden of chronic diseases and sequelae to NTDs. Only through such debates will the estimations of the burden of disease further improve.

There are two major NTDs linked to blindness—trachoma and onchocerciasis. For trachoma, the DALYs only consider disease due to active infection and do not consider blindness that exists even after removal of the infection. For onchocerciasis, the DALYs do not consider the excess mortality due to blindness [23] and likely underestimate the effects of onchocercal skin disease. Furthermore, the onchocerciasis estimates have ignored the burden in the Americas and low-endemic African countries, which may now be relatively small compared to the burden in Africa but was not negligible in 1990. Hence, in both instances the disease burdens from blinding NTDs may represent underestimates.

Finally, in terms of YLDs, important “newcomers” on the GBD scene were the food-borne trematodiases, cysticercosis, and echinococcosis, which must now be recognized as important causes of global disability. Still, no deaths were ascribed to either clonorchiasis or opisthorchiasis (two of the key food-borne trematode infections) in the GBD 2010, despite the strong evidence base linking these liver fluke infections to cholangiocarcinoma in Southeast Asia and elsewhere [36], [37]. Similarly, the YLLs from cysticercosis are most likely underestimated. Indeed, a recent systematic review of the literature showed the proportion of neurocysticercosis patients under care who died during their follow-up could vary from 0.9% to 18.5% [27]. Mostly due to a lack of available data on a global scale, the current estimate for cysticercosis is limited to its role in epilepsy in endemic countries and does not yet include the role of this infection in causing severe chronic headaches and hydrocephalus, depressive disorders, stroke, gliomas, and other neurological sequelae [24].

Among the killer NTDs, almost all of the DALYs due to diseases such as rabies, dengue, and African trypanosomiasis resulted from YLLs, and practically no disability was associated with nonlethal effects from these conditions (YLDs) (Figure 1). However, for dengue, considerable evidence now points to a potentially higher percentage of DALYs due to YLDs (∼25%) as a result of underreporting of nonfatal cases [38], [39]. Similarly, for leishmaniasis the DALY estimates mostly considered the large number of deaths resulting from visceral leishmaniasis but included virtually nothing from the disability of cutaneous leishmaniasis. This finding is a debatable point given the evidence linking disfiguring cutaneous (and mucocutaneous) leishmaniasis on the face to stigma and its impact on girls and women [40]. In addition, for African trypanosomiasis there is also a long-term disease burden resulting from nonfatal consequences, including those suffered by survivors who are eventually treated [41]. Chagas disease was one of the important NTDs whose DALYs were roughly equally distributed between YLDs and YLLs.

## Trends

**Figure 2:**
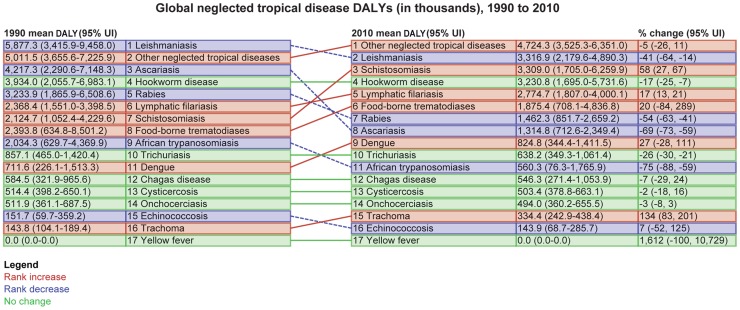
Global trends in DALYs from NTDs, 1990 to 2010. *Estimation of percent (%) change is not from the means. Each metric in this figure is estimated on 1000 times in the modeling process, and then causes that have a high degree of uncertainty in their draw estimates can have skewed % change results. Abbreviations: UI, unit interval.

Figure 2 depicts the ranking of the different NTDs in 1990 as compared to 2010. Although the estimates for both years stem from GBD 2010 and are therefore extrapolated by using the same methodology, they must be interpreted with great care given that the accuracy of the underlying data may have changed through time, with more accurate diagnostic tests becoming available in recent years. The survey locations for frequency data may also have varied between the two periods.

As shown in Figure 2, ascariasis exhibited the largest decrease in DALYs, possibly as a consequence of deworming and socioeconomic development, although it could also reflect the fact that many follow-up studies may have been conducted in areas where such control programs took place. In addition, ascariasis exhibited the greatest decrease in rank, whereas the rankings for trichuriasis and hookworm disease remained constant. The basis for this difference among the intestinal nematode infections is not known, although it may be related to the differential susceptibility of the different helminth species to benzimidazole anthelmintics [42]. It is anticipated that helminth control through mass drug administration and improved access to clean water and sanitation may alter epidemiologic patterns and disease prevalence in the coming years [43].

African trypanosomiasis and rabies (and some other NTDs) were also greatly diminished, the former possibly due to increased access to public health control in association with the resolution of some civil and international conflicts in sub-Saharan Africa [44]. In contrast, DALY estimates for schistosomiasis, lymphatic filariasis, and trachoma appear to have increased over the past 20 years. The underlying bases for these increases include population growth, ecological transformations (e.g., construction of large dams and irrigation systems), and possibly increased surveillance, although it is anticipated that as integrated parasitic disease control and preventive chemotherapy initiatives progress and access to clean water and sanitation increases, we should witness a reduction in several of these disease burden estimates in future years [43]. For dengue, urbanization and increases in global commerce and travel contribute to the emergence of this important disease [45], [46], but increased access to diagnostic tools may also play a role. Since the publication of the GBD 2010, a new estimate suggests that as many as 390 million cases of dengue infections now occur annually [47], more than three times the previous estimates by the WHO.

## Geographic Distribution

**Figure 3:**
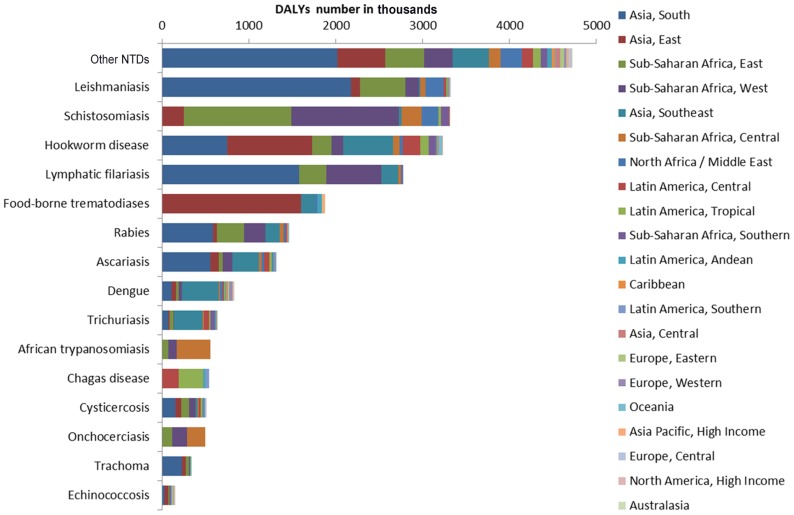
DALYs: Number by disease and for the 21 regions in 2010 (in thousands).

Comparison in the geographical distribution of NTDs must also be conducted with great care since the quality and quantity of data available will depend on where epidemiological studies have been conducted. In addition, within each country, the reported country-level DALYs may be based on surveys conducted specifically in areas where an infection is known to be endemic, which may increase their relative importance as compared to countries where surveys have not been conducted due to a lack of funding or have been conducted in both endemic and nonendemic areas of the country. It is also important to emphasize that many NTDs are of local or of focal importance, often affecting marginalized populations who may not be recognized as national priorities [48]. However, keeping these limitations in mind, the GBD 2010 suggests that there exists an extensive geographic distribution of the NTDs, with sub-Saharan Africa representing the highest DALY rate per 100,000 individuals from NTDs—in part because of their high prevalence together with coinfections that result from hookworm disease, schistosomiasis, onchocerciasis, and African trypanosomiasis [1]. Oceania also has a disproportionate share of NTDs (especially from hookworm disease in Papua New Guinea), as does Southeast Asia, South Asia, and tropical Latin America [1]. Overall the largest (net) number of DALYs from NTDs occurs in Asia (Figure 3). It has been noted that the largest number of cases of many of the high-burden NTDs actually occur in the large emerging-market Asian countries such as China, India, and Indonesia, as well as other countries of the group of 20 (G20) nations [49].

In many Latin American countries, Chagas disease is the predominant NTD. Exceptions are several countries where either intestinal nematode infections predominate (e.g., Colombia, Ecuador, and Venezuela) or Chagas may be underreported, and Haiti and the Dominican Republic, where dengue is the largest source of DALYs. In Bolivia and Peru, food-borne trematodiases rank closely with Chagas disease as the leading NTDs, while emerging information about Chagas disease in the United States [50] may eventually make it an important NTD there as well. Schistosomiasis is the predominant NTD among sub-Saharan African countries, except in selected nations where leishmaniasis (e.g., Sudan), African trypanosomiasis (e.g., Democratic Republic of the Congo, Central African Republic, and Chad), onchocerciasis (e.g., Cameroon), lymphatic filariasis (e.g., Senegal and Guinea-Bissau), intestinal nematode infections (South Africa, Botswana, and Namibia), or rabies (Niger) rank higher. In the Middle East, leishmaniasis is an important NTD, while rabies is the predominant NTD in Afghanistan. In Asia, leishmaniasis is the leading NTD in India; food-borne trematodiases predominate in China, North Korea, and Japan; and intestinal nematode infections are the leading NTDs in much of Southeast Asia (with the exception of dengue in Lao PDR) and Papua New Guinea.

## Missing in Action

There remain some important NTDs for which there are no or limited published disease-burden estimates. These include strongyloidiasis [51], toxocariasis [52], and loiasis, which are among the most common parasitic nematode infections worldwide, as well as toxoplasmosis [53], an important maternal-child protozoan infection that has recently been linked to schizophrenia in immune-competent people and to issues of mental health; leptospirosis, a major bacterial infection; and podoconiosis, a noninfectious condition. In order to estimate the burden subsumed and named as “other NTDs”, the respective cases of death were modeled by using a Cause of Death Ensemble model (CODEm) tool [2], [54], and then the ratio of YLLs to YLDs as derived from the rest of the NTDs was applied to extrapolate the respective YLDs.

## Concluding Statements and Future Directions

An important overriding conclusion of the GBD 2010 is the apparent global shift away from communicable to noncommunicable diseases (NCDs) [1], [55]. Such a conclusion must be tempered by the knowledge that many NTDs are actually underlying causes of the so-called NCDs. In 2008, several NCDs were described, including cancer, cardiovascular disease, and liver disease, that result from chronic long-standing NTDs or from past infections with NTDs such as cysticercosis [56]. With regards to cancer, a new review has identified a substantial burden that can be attributed to infectious diseases [57]. These estimates suggest that, globally, 16% of cancers are caused by underlying infectious agents, and in some developing regions such as sub-Saharan Africa, almost one-third of cancers are caused by infections [57]. In terms of the NTDs, it is known that *Schistosoma haematobium* (the cause of urogenital schistosomiasis) and three of the major liver flukes—*Opisthorchis viverrini*, *O. felineus*, and *Clonorchis sinensis*—are potent carcinogens responsible for a substantial but largely unknown burden of bladder cancer and cholangiocarcinoma, respectively [36], [58], [59]. The burden of cardiovascular disease attributed to NTDs has been recently summarized [60], as have some interesting links between NTDs and chronic liver disease [61] and between onchocerciasis and epilepsy [62]. As new information is obtained, the number of NCD YLLs and YLDs attributed to NTDs will almost certainly increase.

The GBD 2010 is not intended to be the final word on the global disease burden resulting from NTDs. Additional research is needed for almost all of the NTDs, and it is expected that as new information becomes available it can be incorporated into new DALY estimates. For example, the annual number of officially reported dengue cases in eight endemic countries in the Americas and Asia (574,000) is almost three times the episodes estimated by GBD 2010 (Table 4) [63]. Other important examples include the nonlethal consequences of African trypanosomiasis, dengue, and leishmaniasis that will add a larger YLD component to disease burdens for these conditions, as well as the deaths that result from cysticercosis, food-borne trematodiases, hookworm disease, onchocerciasis, and schistosomiasis, among others, which will add YLLs. The GBD 2010 will be updated regularly, which might also allow epidemiologists and policy makers to observe spatiotemporal and presumably declining trends in ascariasis, African trypanosomiasis, lymphatic filariasis, onchocerciasis, trachoma, and possibly other NTDs as a result of preventive chemotherapy and other control interventions. In so doing, a sincere hope is that the GBD 2010 can become a living and breathing document with the flexibility to adapt and change and can ultimately resolve discrepancies and controversies on the true disease burden resulting from NTDs and diseases, injuries, and risk factors.
